# Reasonableness of Enriching Cow’s Milk with Vitamins and Minerals

**DOI:** 10.3390/foods11081079

**Published:** 2022-04-08

**Authors:** Dagmara Woźniak, Wojciech Cichy, Małgorzata Dobrzyńska, Juliusz Przysławski, Sławomira Drzymała-Czyż

**Affiliations:** 1 Department of Bromatology, Faculty of Pharmacy, Poznan University of Medical Sciences, Rokietnicka 3, 60-806 Poznan, Poland; dagmara.wozniak94@gmail.com (D.W.); mdobrzynska@ump.edu.pl (M.D.); jotespe@ump.edu.pl (J.P.); 2 Department of Cosmetology, Faculty of Health Sciences, The President Stanisław Wojciechowski State University of Applied Sciences in Kalisz, Nowy Świat 4, 62-800 Kalisz, Poland; wbcichy@wp.pl

**Keywords:** fortification, calcium, vitamin D, vitamin A, iron, magnesium

## Abstract

Milk is an exceptional nutritional product that has been used for many millennia in human nutrition. Milk is a source of many valuable nutrients, including calcium, vitamin B, an especially significant amount of vitamin B2 and fat-soluble vitamins, such as A, D and E. Milk is an attractive product for fortification as it has a high nutritional density in a small volume and a relatively low price. Research shows positive health effects of drinking milk and consuming dairy products. Even more health benefits can be obtained from consuming fortified dairy products. A literature review, current nutritional recommendations, medical recommendations and an analysis of the market situation all recommend introducing milk enriched with minerals in combination with vitamins to the market. This concept corresponds to the current market demand and may supplement the missing and expected range of fortified milk and the correct number of recipients.

## 1. Introduction

Milk is an exceptional nutritional product that had been used for many millennia in human nutrition [1,2]. Milk was essential for survival for tribes living in different climates where there was a scarcity of fresh plant food in winter. As a result, milk and its products are considered among the most versatile products in terms of nutritional value and—as shown by many years of scientific research—are an essential and valuable component of food for most people in the world [1,3,4].

Milk is composed of about 87% water. It contains, on average, 3.5% protein, about 5% lactose, a variable amount of fat, from 0.5% (in skimmed milk) to 3–4% (in whole milk) and minerals (1.2%, mainly calcium and phosphorus) [3,5,6].

Milk contains calcium, which is essential for bone building. Thus, it is a critical component of the diet throughout the life of a human being around the world. In addition, an important component of milk for disease prevention is conjugated linoleic acid (CLA), which has an anticancer effect [7,8].

Due to milk’s nutrient profile, it is considered a protective food in which the concentration of essential nutrients is high in relation to its energy value [9]. Milk lacks soluble fibre and is poor in omega-3 fatty acids and phytosterols. However, it can serve as a carrier for nutrients and bioactive food ingredients [9].

Milk and its products can protect the body against protein and caloric deficiencies. Ensuring access to high-quality food products, including modified or fortified food products, is essential for every age group. Milk contains a significant amount of easily digestible, wholesome protein with an exemplary qualitative and quantitative composition of exogenous and endogenous amino acids. In addition, milk’s nutritional value is enriched with highly digestible calcium, vitamin B, an especially significant amount of vitamin B_2_ and fat-soluble vitamins, such as A, D and E [1,2,6,10].

Milk contains a group of proteins that prevents calcium precipitation and therefore facilitates the absorption of this component by the body. This phenomenon determines the recognition of milk and milk products as the best source of calcium. It is also one of the reasons why nutritionists adhere to the recommendations for milk consumption. The presence of easily digestible calcium in milk (as in any other food product) is used by the body for the proper development of the skeletal system (growth) and in many metabolic processes of all body cells [1,2,6,10]. While protein, fats and vitamins in milk can sometimes be replaced with other food products, its calcium cannot be replaced with anything else. Therefore, growth, its pace and harmony are fundamental issues that should be supported in children and adolescents [1,2,6,10].

Dietary rules define the exact daily norms of milk consumption for all age groups of healthy people and disease states. In addition, the standards are intended to ensure an adequate supply of valuable nutrients, primarily calcium, for developing organisms (children and adolescents) [11]. Therefore, milk and milk products are included in all nutritional recommendations for the overall population.

The content of vitamins A, D and E in skimmed milk is reduced, but such milk still provides the same amount of protein, calcium and vitamins B as whole milk. When recommending low-fat milk to adolescents and adults, we are primarily guided by the desire to limit animal fats in the diet, especially saturated fatty acids, which have anti-atherosclerotic and anti-cancer effects [12,13]. These recommendations mainly apply to milk fat in the form of butter, and this is dictated by the high content of saturated fatty acids in milk fat, such as: lauric, myristic, palmitic and cholesterol. In diseases related to malnutrition, such as cystic fibrosis, using regularly, appropriately selected preparations of pancreatic enzymes and consuming 1.5% or 3.2% milk or its products, we do not have to worry about problems related to digestion and absorption of fats [14,15,16].

The authors of this paper conducted their research on 363 Polish children aged 1–3 years old. The analysis of their food diaries showed significant nutritional irregularities, including a considerable amount of protein and simple sugars and too little fats, vitamin D, iron and calcium (preliminary study, data still unpublished). In the opinion of doctors and nutritionists, milk and its products should be included in everyone’s daily diet. It is difficult to adequately meet the recommended consumption standards without them, especially for calcium and riboflavin (vitamin B_2_) [17].

Thus, this review aimed to conclude whether or not the fortification of milk with vitamins and minerals is reasonable.

## 2. Market Analysis

Average milk consumption in the European Union (EU) in 2020 was around 65 kg per person [18]. In many countries, the EU average is higher than reality. For example, milk consumption in the Netherlands in 2020 was only around 38 kg per capita. Although the EU is the world’s leading producer of milk and many Europeans drink it on a regular basis, India consumes more than twice as much milk as the EU [18].

According to the information on the official website of the European Commission, the European Union is a significant producer of milk and dairy products covered by the Common Organization of the Markets in Agricultural Products (WORR) [18].

Milk production occurs in all EU countries and accounts for a significant part of the value of EU agricultural production. The total EU milk production is estimated to be around 155 million tonnes per year. The main producers are Germany, France, Poland, The Netherlands, Italy and Ireland [18,19,20]. They are collectively responsible for nearly 70% of the EU production [18].

In recent years, the number of dairy cattle in the EU has decreased, while the milk yield per cow has increased. In 2020, around 20 million cows were raised in the EU, with each cow yielding an average of 7300 kg of milk [18].

In recent years, the milk market in Europe has stabilised to some extent [21]. However, producers’ shares are stable, as is the sales structure according to the type of milk, fat content and packaging. Since 2008, the annual changes in milk sales have been slight. In 2009, there was only a 1% increase in sales [21]. The weakness of the European milk market is its still marginal share of value-added products, such as: low-lactose milk, milk with vitamins, minerals or omega-3 acids [21].

In recent years, most dairy products’ consumption structure has been decreasing. However, the most disturbing factor is the systematic, significant decrease in milk consumption and its consequences. This phenomenon raises many concerns for the health of society. Moreover, it entails a nutritional deficiency of one of the essential nutrients supplied by this group of products, such as calcium [1,2,3,4,5,6].

The phenomenon of reduced milk consumption is a major concern for healthy populations and future generations’ development. Thus, encouraging milk consumption and its products seems reasonable, given that proper nutrition requires each child and adult to consume two glasses of milk or the equivalent amount of yoghurt or kefir daily, as well as one or two slices of cheese [11,21].

Meeting the norm for calcium in Poland may be even below 50%, depending on the age group [22]. It also results in a reduced intake of protein and B vitamins, of which milk and milk products are a significant source in the daily nutrition of the population [11,22]. Therefore, it becomes necessary to introduce and maintain milk with minerals (especially calcium) and vitamins (especially vitamins A and D) on the market [10,23].

According to the Codex Alimentarius (Codex Alimentarius), food fortification or enrichment is the addition of micronutrients to foods, whether or not they are usually contained in the food, to prevent or correct a demonstrated deficiency [24].

Milk is an attractive product for fortification, as it has a high nutritional density in a small volume and at a relatively low price [25]. In addition, modern food processing methods mean that properly prepared milk can be stored for several months [25].

The above comments on the possibility of producing milk with added value (in the analysed case with calcium, vitamins D and A) are also present in observed trends in the milk industry [17,21]. One market trend that has been observed is the decline in demand for plain, pure milk and the emerging trend of flavoured milk consumption. The second trend is shifting demand for milk with a medium and higher fat content (1.5% and 3.2%). Milk with a 0.5% and 1.5% fat content is gradually losing its share in the sales structure [21]. This is because consumers perceive these types as having no proper taste. Thus, the concept of producing 1.5% and 3.2% calcium-enriched milk with vitamins A and D should correspond to the current market demand. It may complement the missing but expected range in the milk market and find the proper recipients [17,21].

## 3. Consumer Demands

An additional argument in favour of the production of innovative milk with added components is that there have been successful attempts to market dairy products that meet the requirements of functional products worldwide [26,27,28]. These products also require promotional activities for the broad population consuming enriched milk and milk products. However, in Poland, incomplete data suggest that consumers already consider milk to be healthy enough and do not necessarily understand the need for its further fortification. Therefore, the gradual expansion of the enriched milk segment should be considered natural [21].

Given the theoretically foreseeable increase in demand for various forms and ingredients added to enriched milk, functional milk products might become a vital element of the market in the future. These products are targeted towards children, teenagers and people over 40–50 years of age. Fortifying milk with omega-3 fatty acids sets an excellent example of their positive role in developing and maintaining normal intellectual functions in children and adolescents [27].

For people over 40 years, milk enriched with phytosterols is promoted, protecting the heart and preventing atherosclerosis [29,30]. Another particular group of functional milk products that should be mentioned is milk enriched with antioxidants, such as coenzyme Q10. All types of fortified milk contain vitamins and minerals. Based on the above analysis, the concept and justification for supplementing milk with 1.5% and 3.2% fat content with calcium in combination with vitamins A and D, both of which are often found to be deficient in European populations, are entirely correct [31,32,33].

## 4. Minerals and Vitamins in Milk

Milk is a source of many minerals and vitamins [1,2,6,10,17]. The content of nutrients in various types of drinking milk is presented in Table 1 and Table 2.

There are two main types of cow milk on the market: pasteurised and ultra-high temperature (UHT). Heat treatment of milk completely destroys pathogenic microflora and minimises the number of saprophytic microorganisms that can cause spoilage. The production of pasteurised milk is carried out using a heat treatment involving the application of high temperature for a short period—usually within 15 s at a temperature of 72 °C [35]. It improves the safety and extends the shelf life of a product. Pasteurised milk almost entirely preserves the nutritional properties of raw milk while ensuring safety and health. Heat treatment moderately reduces the concentration of some vitamins, especially vitamins C and B-complex, but not fat-soluble vitamins. Loss of vitamin C amounts to around 0–10%, while B-complex vitamins from 1% for vitamin B_2_ to 20% for B_9_ [35,36,37]. Moreover, it does not affect the fatty acid profile [38]. Due to its good nutritional quality and hygienic safety, pasteurised milk is recommended for all milk for human consumption by the Food and Agriculture Organization and the Food and Drug Administration (FDA) [39,40].

Regardless of the method of heating, the content of protein, fat, lactose and minerals is practically unchanged [41]. The process of heating milk with the UHT method increases the loss of vitamin B by 10 to 20% compared to raw milk. In the case of pasteurised milk, when the storage time is from a few days to 21 days at a temperature ranging from 1 to 8 °C, the loss of vitamins is not significant. In UHT milk, the loss of some nutrients is significant. The following examples show the loss of essential nutrients [42]:

- Vitamin A (30% loss during the storage period of five–six weeks);
- Vitamin B_6_ (almost complete loss after several weeks of storage);
- Vitamin B_12_ (almost complete loss after prolonged storage);
- Vitamin C (almost complete loss up to two weeks of storage).

Table 3 shows the comparison of vitamin losses between pasteurised and UHT milk.

### 4.1. Calcium

Calcium is essential for the proper development of the skeletal system and many metabolic processes in all body cells, such as nerve and muscle cells. Calcium serves as a regulatory and building element in the body. Calcium salts are part of the skeleton and teeth, ensuring adequate strength and durability. Calcium is essential for the maintenance of normal blood pressure and nerve conduction. Research shows that calcium reduces fat cell growth, thus preventing obesity. When it is deficient in the blood, the body extracts it from the bones. Calcium ions participate in the formation and maintenance of tissue excitability and the conduction of nervous tissue stimuli. They also support the release of neurotransmitters and maintain the resting tone of muscles (including the heart muscle) and the contractility of nerve fibres. Calcium is an activator of blood coagulation processes involved in forming haemoglobin and maintaining the acid–base balance. Calcium deficiency in the body may limit or prevent the formation and progression of these life processes. Vitamin D, lactates and citrates support calcium absorption in the digestive tract. However, it is difficult to absorb calcium in the presence of aluminium compounds, phosphates (added to many products and carbonated drinks), excess magnesium, potassium and strontium [10,43,44].

The recommended daily allowance (RDA) for calcium varies by age: 700 mg/day up to the age of three, 1000 mg/day for children aged four–nine, 1300 mg/day for adolescents and 1000 mg/day for adults. For people over 75 years, due to limited absorbability and the high need, calcium is recommended at 1200 mg/day [11].

The coverage of the daily requirement (RDA) for calcium in the diet of Poles is insufficient, possibly even below 50%. That is mainly due to the low consumption of dairy products [22].

Even 70% of dietary calcium in the diet comes from milk and dairy products, mainly cheese, and only approximately 16% comes from some green vegetables and dried fruit, while 6–7% is provided by mineral water. Calcium from plant products is poorly absorbed due to the fibre and oxalic acid in vegetables and phytic acid in cereal products. Therefore, the best sources of calcium are skimmed milk, yoghurt and semi-skimmed cheese, as the lactose in them supports the absorption of calcium [11,43,44].

### 4.2. Vitamin D

Vitamin D includes vitamin D_1_ (calciferol), D_2_ (ergocalciferol) and D_3_ (cholecalciferol). The source of vitamin D_1_ is fish oil. D_2_ is produced in plants exposed to ultraviolet rays. In contrast, vitamin D_3_ is formed in the skin of humans and animals. Under the influence of sunlight, the body can produce vitamin D_3_ itself. It transforms 7-dehydrocholesterol in the human skin (provitamin D_3_ of endogenous origin) into cholecalciferol. Vitamin D is closely related to converting calcium and phosphorus—the two main bone-forming substances [45,46,47,48].

Vitamin D increases the absorption of calcium and phosphate from the gastrointestinal tract. It promotes ossification and bone mineralisation, optimises bone mineral density, reduces the risk of falls and fractures and prevents the formation of cramps in the muscles. It is essential for the proper functioning of the muscular, nervous, immune and endocrine systems. Active metabolism of vitamin D regulates cell functioning and differentiation and may exert immunomodulatory and antiproliferative effects. The presence of vitamin D prevents various types of cancer, such as non-Hodgkin’s lymphoma, colon cancer, breast cancer and prostate cancer. It regulates insulin secretion, soothes and helps to prevent skin inflammation. It increases immunity by affecting the bone marrow cells that produce defence cells (monocytes) and improves auditory function by involving the ossicles [45,46,47,48].

Vitamin D is one of the most common vitamins in the body. Therefore, even young children should be supplemented with this vitamin, especially in winter. Its deficiency in children and infants causes rickets (a bone calcification disorder) and late rickets in older children and adolescents. In adults, vitamin D deficiency results in bone structure disorders, such as: osteomalacia and bone thinning, skin inflammation, myopia, neurotic ailments, insomnia, conjunctivitis, decreased immunity, hearing impairment and weakening and loss of teeth [45,46,47,48,49].

From a nutritional and clinical point of view, it is essential to understand the symptoms of vitamin D overdose, which include: nausea, diarrhoea, weight loss, loss of appetite, persistent fatigue, drowsiness, sweating, eye diseases, itchy skin, higher risk of atherosclerosis and urolithiasis disorders, migraines, muscle, joint and toothaches, frequent urination, arrhythmia and delay in physical development during the growing period [50].

However, overdose conditions are much less common than deficiencies. For the health of the whole organism, especially the bones and teeth, it is important to cover the daily requirement for vitamin D in an optimal dose. The guidelines focusing on bone health and metabolism recommend a target 25(OH)D concentration of 20 ng/mL (50 nmol/L) and daily vitamin D doses of 400–800 IU, depending on the age. The guidelines concerning pleiotropic effects of vitamin D recommend a target 25(OH)D concentration of 30 ng/mL (75 nmol/L) and vitamin D doses ranging between 400 and 2000 IU/day depending on age, body weight, ethnicity and diseases [11,51].

According to the current nutritional recommendations in Poland, the prevention of vitamin D deficiency should start with the youngest children. According to European Vitamin D Association (EVIDAS) and other scientific societies and National Consultants, during infancy and up to six months of age, children should receive 400 IU/day of vitamin D supplementation and 400–600 IU/day from six months to one year of age, depending on the daily amount of vitamin D taken with food [50,51]. Based on body weight and the dietary vitamin D intake, 600–1000 IU/day is recommended for children up to 10 years and 800–2000 IU/day for adolescents. An adequate intake (AI) of vitamin D is 10 μg of cholecalciferol/day for infants and 15 μg cholecalciferol/day for children and adults [11,50,51].

The study ‘Comprehensive assessment of the nutrition of children aged 5–36 months in Poland’, conducted in 2010–2016 by the Institute of Mother and Child in Warsaw, showed vitamin D deficiency in the diets of the studied children. The supply did not meet the Consultants of the National Board of Paediatrics’ recommendations for the prevention of rickets and osteoporosis. Ninety-four per cent of children aged 1–3 did not receive adequate vitamin D from their diet. On the other hand, the content of B vitamins (B_2_, B_6_, B_12_ and PP) and vitamin C in the average food ratio exceeded the norms by two or even three times [52].

According to Cashman et al. [32], vitamin D deficiency is evident throughout the European population at concerningly prevalence rates, requiring action from a public health perspective. The map of vitamin D status in different countries (based on 107 studies involving more than 630,000 subjects) revealed that one out of every eight people was at risk of vitamin D deficiency (even in sunny regions) [5,32].

In some countries (e.g., the USA, Canada, Finland, Poland), it is assumed that selected foods enriched with vitamin D can be used to compensate for deficiencies. They often include milk, margarine, dairy products, breakfast cereals or cereal products. In the USA, in recent years, fortification with calcium and vitamin D has been used for juices and drinks [53].

In Poland, it is obligatory to enrich foods with vitamin D, such as margarine and butter–oil mixtures (maximum amount not more than 7.5 µg/100 g) and milk for infants (starting and following), as well as baby formula [54]. The natural content of vitamin D in cow’s milk is low, ranging from 0.1 to 1 μg/L in full-fat milk [5]. Vitamin D is fat-soluble, so its products should be enriched with fats for better absorption or added to products such as milk containing 1.5% or 3.2% fat [17,21]. Various products on the national market are already enhanced by manufacturers voluntarily, such as breakfast flakes, milk and milk drinks, yoghurts, homogenised cheese, milk desserts, juices, instant cocoa drinks and plant-based drinks (milk substitutes) [53].

Itkonen et al. [55] showed that vitamin D-fortified fluid milk products contribute to vitamin D intake and 25(OH)D status. Milk products contribute 28–63% of vitamin D intake in countries with a national vitamin D fortification policy covering various fluid milk products (Finland, Canada, United States). This contribution is much lower or negligible in countries without a fortification policy or fortification concerning only some dairy products (Sweden, Norway) [55].

In a randomised controlled trial in Denmark, the effect of vitamin D fortification in milk and bread was investigated for 782 children and adults (4–60 years) recruited from 201 families. The intervention lasted six months. Median vitamin D intakes (habitual diet plus fortified products) were 9.4 mg/day (6.5, 12.3 mg/day) and 2.2 mg/day (1.5, 3.0 mg/day) in fortification and control groups, respectively. The final 25(OH)D concentration was significantly higher in the study group than in the control group (*p* < 0.001) [56].

Sixty-three Chinese women (>55 years) received two servings of either a high-calcium vitamin-D-fortified milk or a control drink for 12 weeks. Serum 25(OH)D levels improved significantly (33.13–39.49 nmol/L) in the study group, while in the control group they remained similar (29.27–28.21 nmol/L). Fortified milk consumption significantly improved vitamin D status and reduced bone turnover [57].

In New Zealand, 181 healthy toddlers aged 12–20 months participated in a partially blinded, randomised 20 week trial [58]. The effect of feeding red meat or fortified toddler milk (vitamin D-fortified milk and micronutrient-fortified milk) on the iron, zinc, iodine and vitamin D status was measured. After the intervention, mean serum 25(OH)D concentrations significantly increased in the groups receiving milk. The prevalence of having too low serum 25(OH)D < 50 nmol/L differed between groups. It remained relatively unchanged in the meat group (*p* = 0.24) and substantially decreased in the vitamin D-fortified whole-milk group (*p* = 0.017 compared to the meat group) as well as the micronutrient-fortified milk group (*p* = 0.008 compared to the meat group). The researchers concluded that regular consumption of vitamin D-fortified milk (a mean intake of nearly 4 mg/day) effectively achieved adequate year-round serum 25(OH)D for most children [58].

The US data also show that as milk consumption declines, so does vitamin D intake [59,60]. The history of the regulation of milk fortification with vitamin D dates back to the 1930s in the USA [59,60].

### 4.3. Vitamin A

Fat-soluble vitamin A is stored in the body, primarily in the liver. Experts distinguish between two forms: vitamin A, also known as retinol, which occurs only in animal products, and provitamin A, also known as beta-carotene, which is found in both animal and vegetable foods [61].

Vitamin A plays an essential role in the development and differentiation of body cells, especially epithelial cells and bone tissue. It is indispensable in the vision process, constituting an integral component of the retina of the eye. It also influences spermatogenesis, placenta development and embryonic growth. The optimal supply of this substance ensures the proper activity of the immune system and strengthens both its humoral and cellular components. Vitamin A is responsible for the appropriate functioning of the mucous membranes of the eyeball, digestive, respiratory and genitourinary systems and the course of the IgA-dependent response [62,63,64]. According to some research, vitamin A is one of the most effective substances in delaying ageing. It accelerates the epidermis renewal by influencing the synthesis of proteins, metabolism and cell division. Vitamin A improves the structure of the stratum corneum and enhances the protective function of the epidermis. As a result, water loss through the skin is reduced. In the dermis layers, it stimulates the production of collagen and elastin. It causes the reaction of transforming low-activity fibroblasts into cells with relatively high collagen production, improving skin firmness, elasticity and hydration. Vitamin A also protects the resulting collagen against degeneration by stimulating the reconstruction of reticulin fibres and the formation of new blood vessels in the papillary layer of the dermis. It has a significant impact on the water–fat balance, thus reducing the roughness and peeling of the skin [62,63,64].

Vitamin A prevents ‘night blindness’ (deterioration of vision after dark), improves visual acuity and aids in the treatment of many eye diseases (participates in the synthesis of pigments in the retina of the eye). In addition, the vitamin supports the proper functioning of the immune system and the defence of the respiratory system (as it ‘seals’ mucous membranes). Furthermore, it shortens the duration of infectious diseases, strengthens the epidermis and dermis (prevents drying and keratosis) and helps in the removal of age-related skin discoloration. The application of vitamin A in treating acne, wrinkles, lichenoid changes, emphysema, hyperthyroidism, abscesses, boils and open ulcers is widely recognised. Moreover, vitamin A is essential for developing bones, teeth and proper functioning of the genital organs [62,63,64].

Sources of vitamin A in food are apricots, pumpkin, watermelon, melon, asparagus, broccoli, carrots, chicory, kale, lettuce leaves, spinach, liver, eggs, milk and preserves. The desired fat percentage in milk should facilitate the proper dissolution of vitamin A (retinol) and ensure optimal systemic utilisation [11,34].

According to Hombali et al., vitamin A deficiency is a significant public health issue in many low- and middle-income countries. It mainly affects young children, women of reproductive age and pregnant women [65]. The World Health Organization (WHO) estimates that night blindness affects 5.2 million pre-school-aged children and nearly 10 million pregnant women worldwide [66].

Experts point out that the consumption of milk and dairy products enriched with vitamin A may positively affect the functioning of the immune system [67]. This is especially important in the era of the COVID-19 pandemic, which causes severe respiratory tract infections [68].

Vitamin A fortification is optional for whole milk. However, if added, the concentration must not be less than 2000 IU per quart. In the United States, vitamin A is added as synthetic retinyl palmitate [69].

Vitamin A (microencapsulated form) added externally is more stable than the inherent vitamin A present in milk. However, iron-fortified milk may have its downsides. Research showed that loss of vitamin A (%) was significantly greater (*p* < 0.05) in iron-fortified milk, and the iron may participate in accelerating the degradation of vitamin A. Vitamin A is light-sensitive and thus rapidly degrades, especially in skimmed milk [70,71].

A literature review, current nutritional recommendations, medical recommendations and market analysis justify the introduction of calcium-enriched milk with vitamins A and D to the market. This concept corresponds to the current market demand and may supplement the missing and expected range of fortified milk and the correct number of recipients [72].

### 4.4. Other Nutrients

European food law does not provide milk enrichment with vitamins A or D and therefore does not contain specific provisions on such enrichment [73]. According to European Parliament regulation, milk and dairy products with a lesser fat content can be enriched with calcium and magnesium [73].

Proper selection of mineral carriers is the most critical element in the fortification process of dairy products. The mineral compound must be easily digestible so that the enrichment is effective at preventing deficiencies. Moreover, the fortification process needs to be sufficiently inexpensive so that it does not affect the price of the final product. Calcium and magnesium carriers must be safe for health. They should be characterised by adequate solubility, high chemical stability and heat, and they must not alter the sensory properties of the products. Water-insoluble salts are neutral to casein and may be added to the milk before thermal processing as they do not increase the probability of thermal coagulation of milk proteins. However, their use is associated with the risk of sedimentation in liquid products [22].

Magnesium plays a vital role in protein synthesis, cellular energy production and storage, reproduction, DNA and RNA synthesis and stabilising mitochondrial membranes. It is also required in nerve transmission, neuromuscular conduction, muscular contraction, cardiac excitability, blood pressure regulation and glucose and insulin metabolism. Thus, maintaining an adequate magnesium level is essential for disease prevention and overall health [74].

Low dietary magnesium intake increases the likelihood of developing type 2 diabetes. In addition, magnesium affects calcium metabolism; therefore, its deficiency is considered an important factor in postmenopausal osteoporosis in women [22].

According to Sazawal et al. [75], multiple micronutrient deficiencies are highly prevalent among pre-school children and often lead to growth faltering and anaemia. The authors conducted a double-masked, randomised trial among 633 Indian children aged 1–4 years. The study group received micronutrient-fortified milk (containing additional zinc, iron, selenium, copper, vitamin A, vitamin C, vitamin E). The control group received milk without fortification. The intervention lasted a year. Consumption of fortified milk resulted in a significant decrease in the proportion of underweight children as the weight for age < −2 *Z*-scores (study vs. control: odds ratio  =  0.63 [95% CI from 0.40 to 0.99], *p*  =  0.05). Children in the study group on an average gained more weight (0.23 kg/year; 95% CI from 0.12 to 0.33, *p* < 0.001) and height (0.58 cm/year; 95% CI from 0.33 to 0.82, *p* < 0.001). Furthermore, they had a significant improvement in anaemia and repletion of body iron stores; their mean haemoglobin levels increased by 13.6 g/L (95% CI from 11.1 to 16.0, *p* < 0.001) in the study group compared to the control group. Researchers reported an 88% decrease in the proportion of children with iron deficiency anaemia (95% CI from 80% to 92%, *p* < 0.001) and a significant reduction in the proportion of iron-deficient children (the study vs. control: 68 (29.2%) vs. 164 (70.7%), *p* < 0.001) due to fortified milk consumption [75].

Moreover, fortified milk consumption was associated with an 18% lower incidence of diarrhoea, 26% lower incidence of pneumonia, 7% fewer days with high fever and 15% fewer days sick with severe illnesses [76].

The consumption of fortified milk (with vitamins A, C, D, E, K, B_12_, thiamin and riboflavin) and noodles (enriched with vitamins A, B_6_, B_12_, thiamin, niacin, folate and iron) was associated with a reduced risk of stunting among Indonesian children aged 6–59 months old. The effect was more substantial when both were consumed at the same time. The study indicates the potential benefits of multiple micronutrient fortification for child growth [77].

According to Eichler et al. [78], who performed a systematic review and meta-analysis on 18 trials including 5468 children, iron plus multi-micronutrient fortification is more effective than iron-only fortification in improving haematologic outcomes in micronutrient-fortified milk or cereals. Compared to non-fortified food, iron multi-micronutrient fortification increased haemoglobin levels by 0.87 g/dL (95% CI: 0.57 to 1.16; 8 studies) and resulted in a 57% reduction in the risk of anaemia (relative risk 0.43; 95% CI from 0.26 to 0.71; an absolute risk reduction of 22%; number needed to treat five [95% CI: 4 to 6]; six studies) [78].

As mentioned, milk and milk products have the highest nutritional value. An allergy to cow’s milk protein is the only contraindication to the consumption of milk and dairy products. In the case of lactose intolerance, low-lactose or lactose-free milk can be consumed. Milk with modified lactose content should also be enriched with calcium, iron and vitamins A and D [22,75,76,77,78].

Dairy products enriched with vitamins and microelements are profiled for specific groups of recipients of different ages, who are often exposed to the occurrence of particular diseases. Dairy products enriched with microelements usually contain calcium, phosphorus, magnesium, iron, zinc, copper, manganese, selenium, iodine, chromium, molybdenum and cobalt. In addition, vitamins A, D, C, E and K are also added, as well as, although less often, vitamin K (biotin), pantothenic acid or folic acid. In Poland, functional food with vitamins and minerals is still being developed, although consumer expectations are growing. A significant problem is the under-financing of research and development departments in domestic dairies, the lack of marketing budgets for building consumer awareness and decision makers’ fear about the modest sale volume of new products [22].

An increasing number of products are being fortified (enriched) at considerably high levels, such as supplementing them with a few or even a dozen active ingredients. This group should also include milk enriched with minerals in combination with vitamins [75,76,77,78].

Quality of life issues are increasingly influencing the purchase of dairy products. Therefore, the quality of life is directly related to functional food, fortified food or other technologically advanced products. When introducing new products to the market, producers should consider those that are already on the market. The biggest challenge for the producer may be identifying the target consumer for the new product [22].

## 5. The Comparison of Nutritional Value of Cow’s Milk with Other Mammal’s and Plant-Based Milks

As mentioned above, the analysis of milk production in the EU countries shows that cow’s milk accounts for 96% of the total milk production [20]. Other popular milks are buffalo milk, goat’s milk and sheep’s milk. Other species, such as elk, reindeer, yak, musk ox, llama and alpaca, account for 0.2% of the world milk market [79].

Buffalo milk contains more than twice the amount of cow’s milk fat (7.5 g/100 g vs. 3.3 g/100 g) and thus a higher energy content. Goat’s milk has higher retinol content and higher levels of free amino acids than cow’s milk. However, it also contains low amounts of folate and vitamin B_12_. Sheep’s milk contains more lactose, lower sodium and potassium levels than cow’s milk, but most other minerals are present in higher quantities [79].

Concerns about environmental impact and sustainability, animal welfare and personal health issues result in the growing popularity of plant-based milk products [80]. Commonly available nondairy beverages are derived from coconut, almond, cashew, hemp, hazelnut, oat, rice and soy [81]. Almond milk is higher in sodium compared to other milks and lacks B_12_ and folate. Plant-based milks are low in vitamins D and B_12_ [80]. Most of these beverages are fortified with calcium and vitamin D, but the bioavailability of these substances after fortification is not available [81]. Proper milk choice is crucial for children who follow a strict vegan or vegetarian diet that are poor in vitamin B_12_. There is also not enough evidence that plant-based alternatives are healthier than cow’s milk [81]. None of the plant-based drinks should be used as a substitute for cow’s milk in children below 24 months old. Additionally, some of these beverages contain added sugars and sweeteners. Cow’s milk contains the most protein per serving. Compared to other types of milk, cow’s milk has a better quality [81].

According to Collard and McCormick [82], cow’s milk remains the best fat, protein and micronutrient source. Thus, it is the best choice for vulnerable populations, especially children. It is worth remembering that children aged 1–3 have an increased need for fat in the diet. Observational research suggests that a higher cow milk fat intake is associated with lower childhood adiposity [83]. Milk is also a source of fat, and skim milk may be recommended for children at a higher risk of obesity and familial hypercholesterolemia. However, the research is inconsistent [83].

Cow’s milk consumption is linked to an increased risk for obesity, heart disease and cancer [83]. Those myths may be confusing for people and result in decreased milk consumption [83].

Due to the growing consumption of highly processed food in Europe, often poor in nutritional value, the consumption of milk, including fortified milk, is fully justified in all age groups. Especially since the consumption of convenience food containing phosphates and other functional additives is constantly growing and limits the bioavailability of minerals [84].

The study may be limited because the review is based mainly on cow’s milk. However, for several decades it has been chosen primarily by consumers. Consumer education is essential, as many harmful myths have arisen around the consumption of cow’s milk. The plant-based milk and plant-based dairy products market is viral among consumers. However, as mentioned earlier, plant-based milks are not a satisfactory source of nutrients [81]. Some concern may be raised due to the fact that despite the evident dispositions and potential benefits obtained through a reasonable supplementation of milk with vitamins and nutrients, there is still a lack of official European guidelines. The possible supplementation of dairy products is also noteworthy, which may form the basis for further research.

It is worth noting that speculations regarding the fortification of milk and dairy products have appeared many times in the available literature. However, our work is the first to justify the need to fortify milk based on an analysis of the current dairy market in EU countries, as supported by our research showing nutritional deficiencies in children aged 1–3, emphasising the bioavailability of vitamins and minerals in dairy products and opposing the myths about milk consumption.

## 6. Conclusions

As mentioned earlier, there are great difficulties in meeting the requirements for calcium, iron and vitamins A and D, especially in the diet of young children. There are also reports on the necessity of magnesium and iron supplementation for vulnerable groups, including pre-school children [85]. Taking this into account, it seems necessary and reasonable to enrich milk with minerals and vitamins. It is vital to provide information to parents about the correct time to introduce milk into the child’s diet [79].

More research is needed to examine the long-term outcomes of consuming different types of milk. Care should be taken to ensure proper education of the general population regarding the benefits of milk consumption and the nutritional differences between various types of milk so they make the best individual choice [86].

## Figures and Tables

**Table 1:** Milk market share statistics for 2016–2020 in 27 European Union countries. Adapted from [20].

Product	2016		2018		2020	
	mln Tones	%	mln Tones	%	mln Tones	%
whole fresh cow milk	147.71	96.60	151.27	96.51	154.40	96.40
whole fresh sheep milk	2.99	1.96	2.84	1.81	2.97	1.85
whole fresh goat milk	2.00	1.31	2.36	1.51	2.50	1.56
whole fresh buffalo milk	0.21	0.14	0.27	0.17	0.29	0.18

**Table 2:** Content of selected minerals in mg/100 g of drinking milk. Adapted from [34].

Product	Calcium	Phosphorus	Potassium	Magnesium	Zinc
pasteurised milk 3.5% fat	118	85	138	12	0.32
pasteurised milk 3.2% fat	118	85	139	12	0.32
pasteurised milk 2.0% fat	120	86	141	12	0.32
pasteurised milk 1.5% fat	120	97	141	12	0.37
pasteurised milk 0.5% fat	121	97	141	12	0.40
UHT milk 3.2% fat	113	81	139	12	0.32
UHT milk 1.5% fat	110	92	141	12	0.37
UHT milk 0.5% fat	111	91	141	12	0.40

**Table 3:** Loss of vitamins in pasteurised and UHT milk [42].

Vitamin	% Loss in Pasteurised Milk	% Loss in UHT Milk
folate	5–20	10–20
vitamin A	no significant changes	no significant changes
vitamin B_1_	<10	10–20
vitamin B_2_	<1	no significant changes
vitamin B_6_	<3–5	<10–15
vitamin B_12_	<10	0–30
vitamin C	0–10	<15–25

## Data Availability

No new data were created or analyzed in this study. Data sharing is not applicable to this article.
